# Distribution and antimicrobial resistance of pathogens in oral and maxillofacial infections during and Post-COVID-19 pandemic

**DOI:** 10.3389/fpubh.2026.1805185

**Published:** 2026-04-10

**Authors:** Dandan Li, Zhujun Yu, Cheng Nie, Huamin Li, Jingjing Jiang, Jianli Wang

**Affiliations:** State Key Laboratory of Oral Diseases, National Center for Stomatology, National Clinical Research Center for Oral Diseases, Department of Pharmacy, West China Hospital of Stomatology, Sichuan University, Chengdu, Sichuan, China

**Keywords:** antimicrobial resistance, distribution, oral and maxillofacial infection, pandemic, pathogen

## Abstract

**Background:**

This study investigated the distribution characteristics and antimicrobial resistance patterns of pathogens isolated from patients with oral and maxillofacial infections during and after the COVID-19 pandemic.

**Methods:**

This retrospective study analyzed microbial cultures of specimens obtained from patients with odontogenic and non-odontogenic oral and maxillofacial infections during the pandemic period (January 2020–December 2022) and the post-pandemic period (January 2023–December 2024). Pathogen identification was performed using matrix-assisted laser desorption/ionization time-of-flight mass spectrometry. Antimicrobial susceptibility testing was conducted using the VITEK 2 Compact automated analyzer, the Kirby-Bauer disk diffusion method, and the Etest. Data were analyzed using WHONET (version 5.6) and R software (version 4.2.1). This study was approved by the Medical Ethics Committee of West China Hospital of Stomatology, Sichuan University (Approval No.: WCHSIRB-D-2025-280).

**Results:**

While the microbial culture submission rate remained stable, the culture positivity rate decreased from 20.19 during the pandemic to 18.71% in the post-pandemic period. In odontogenic infections, anaerobic bacteria predominated, with *Viridans streptococci, P. intermedia*, and *P. acnes* being the most frequently isolated species. Notably, clindamycin resistance among *Viridans streptococci* significantly decreased from 93.81 to 82.35%. In contrast, non-odontogenic infections were predominantly caused by aerobic bacteria, most frequently involving *K. pneumoniae, A. baumannii, S. aureus*, and *P. aeruginosa*. Resistance to ceftriaxone in *K. pneumoniae* decreased significantly from 14.91 to 9%, whereas resistance to levofloxacin in *P. aeruginosa* markedly increased from 2.88 to 11.49%. In addition, the proportion of carbapenem-resistant *A. baumannii* (CR-AB) rose from 9.52 to 17.11%.

**Conclusion:**

Following the COVID-19 pandemic, shifts in both the pathogen spectrum and antimicrobial resistance patterns were observed in odontogenic and non-odontogenic infections. These findings reflect an evolving microbial landscape and highlight the need for tailored, etiology-specific antimicrobial strategies in the management of oral and maxillofacial infections. However, as these changes may be attributable to multiple confounding factors, further studies are warranted to establish causality.

## 1 Introduction

Oral and maxillofacial infections encompass a range of infectious diseases that affect the soft tissues of the oral cavity, jaw, face, and neck. Among these, oral and maxillofacial space infections (OMSI) are particularly severe (1, 2). These infections generally originate from either odontogenic sources, including pulp or periodontal tissues (3–5), or non-odontogenic sources, including trauma and lymphadenitis (2, 6). Due to the region's complex anatomy, infections can spread rapidly along fascial spaces, leading to life-threatening complications such as airway obstruction, sepsis, or mediastinitis (7).

Although odontogenic and non-odontogenic infections share clinical features, their distinct microbial compositions dictate different antimicrobial approaches (5, 6). The effectiveness of these treatments is increasingly threatened by the rise of antimicrobial resistance (AMR), which exacerbates the disease burden and impairs clinical outcomes (8). Consequently, while surgical drainage remains the primary intervention, targeted antimicrobial therapy informed by regional epidemiological data is essential for improving efficacy (9).

The global increase in broad-spectrum antimicrobial use during the pandemic has further intensified AMR challenges (10, 11). While surveillance systems in China and other nations monitor pathogens in common conditions like bloodstream or urinary tract infections, data specifically addressing oral and maxillofacial infections remain relatively limited (12–16). Furthermore, systematic analyses of how the COVID-19 pandemic influenced epidemiological trends in this field are currently lacking (17).

Preliminary data from our hospital suggest that common pathogens in this field exhibit high resistance to certain antibiotics. In response, our clinical center has begun strengthening drug-use monitoring and implementing restrictions to optimize antimicrobial stewardship. However, the impact of the pandemic on AMR is complex and varies significantly across different regions and institutions, highlighting the necessity for localized longitudinal analysis (15, 16).

Given that China's national AMR surveillance system does not yet cover oral and maxillofacial pathogens, single-center studies are essential for establishing localized baseline data. This retrospective study compares pathogen distribution and resistance patterns during and Post-COVID-19 pandemic in our hospital. The findings aim to inform empirical antimicrobial therapy, support regional AMR control strategies, and contribute to broader public health policy development.

## 2 Methods

### 2.1 Study design and population

This study included patients with oral and maxillofacial infections who were hospitalized at West China Hospital of Stomatology, Sichuan University, between January 2020 and December 2024. The study period was divided into the pandemic period (January 2020–December 2022) and the post-pandemic period (January 2023–December 2024), with the division based on China's zero-COVID policy adjustment in December 2022. To ensure strict protection of patient privacy, all personal information was anonymized and de-identified. Patients were subsequently categorized into odontogenic and non-odontogenic infection groups based on clinical examinations and imaging assessments. This study was approved by the Medical Ethics Committee of West China Hospital of Stomatology, Sichuan University (Approval No.: WCHSIRB-D-2025-280).

### 2.2 Microbial culture and identification

Clinical specimens, including pus aspirated from abscess cavities and wound swabs collected during surgical drainage, were obtained from patients with oral and maxillofacial infections. All samples were collected under aseptic conditions and transported to the microbiology laboratory within 2 h. Pathogen identification was performed using matrix-assisted laser desorption/ionization time-of-flight mass spectrometry. Antimicrobial susceptibility testing was conducted using the VITEK 2 Compact automated analyzer, the Kirby-Bauer disk diffusion method, and the Etest. Quality control was ensured using standard strains, including *K. pneumoniae* ATCC 700603, *S. aureus* ATCC 25923, *P. aeruginosa* ATCC 27853, and *Candida albicans* ATCC 10231, which were obtained from the Clinical Laboratory Center of the National Health Commission. The results of antimicrobial susceptibility testing were interpreted according to the Clinical and Laboratory Standards Institute (CLSIM100-S33) standards.

### 2.3 Definitions

Duplicate isolates were defined as the same microbial species isolated from the same patient during a single hospitalization episode and were excluded from all analyses. The microbial culture submission rate was calculated as the number of microbial culture samples submitted for oral and maxillofacial infections divided by the total number of all microbial culture samples submitted during the study period. The microbial culture positivity rate was calculated as the number of positive microbial culture samples from oral and maxillofacial infections divided by the total number of microbial culture samples submitted for these infections. The antimicrobial resistance rate was defined as the number of isolates resistant to a specific antimicrobial agent divided by the total number of isolates tested for that agent. Antimicrobial use density (AUD) was defined as the number of defined daily doses (DDDs) of antimicrobial agents consumed per 100 inpatient-days.

### 2.4 Statistical analysis

AMR patterns of pathogens were analyzed using WHONET software (version 5.6). Statistical analyses were performed using R software (version 4.2.1). Appropriate statistical tests, including the chi-square test, Fisher's exact test, or the Mann-Whitney U test, were used to compare parameters between the pandemic and post-pandemic periods. These parameters included microbial submission rates, positivity rates, baseline characteristics, pathogen distribution, AMR patterns, and AUD. Spearman correlation analysis was performed to assess the relationship between meropenem use density and the resistance rates of specific pathogens. Data visualization was primarily performed using GraphPad Prism (version 10.6.0). A *p*-value of less than 0.05 was considered statistically significant.

## 3 Results

### 3.1 Microbial culture submission and positivity rate

The annual microbial culture submission rates from 2020 to 2024 were as follows: 61.38% (3,055/4,977), 55.65% (3,627/6,517), 53.97% (3,817/7,073), 58.37% (5,246/8,988), and 54.14% (5,327/8,839). During the pandemic period, the overall submission rate was 56.55% (10,499/18,567), which slightly decreased to 56.16% (10,573/18,827) in the post-pandemic period (*p* > 0.05). Thus, no statistically significant difference in submission rates was observed between the two periods.

**Figure 1:**
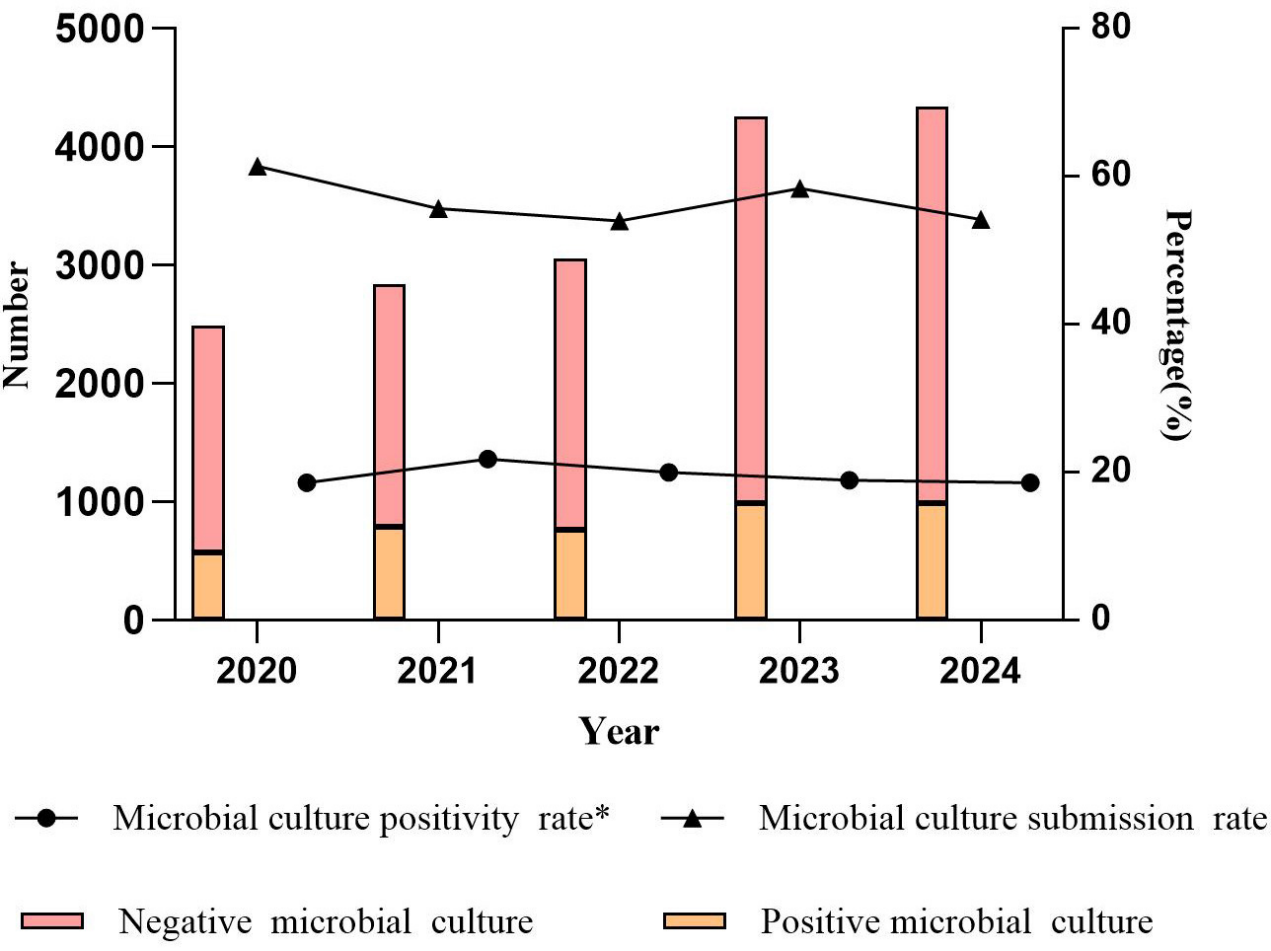
Number of negative/positive microbial cultures, microbial submission and positivity rate; *p < 0.05.

The annual microbial culture positivity rates were as follows: 18.59% (568/3,055), 21.75% (789/3,627), 19.99% (763/3,817), 18.87% (990/5,246), and 18.55% (988/5,327). The positivity rate during the pandemic period was 20.19% (2,120/10,499), which significantly decreased to 18.71% (1,978/10,573) in the post-pandemic period (*p* < 0.05) (Figure 1).

### 3.2 Baseline characteristics of patients with positive microbial culture

The annual numbers of patients with positive microbial cultures from 2020 to 2024 were 301, 311, 294, 349, and 359, respectively. The cohort was predominantly male, with an overall male-to-female ratio of 1.73. The median ages for the pandemic and post-pandemic periods were 47 years (range: 0.42–89) and 49 years (range: 0.67–93), respectively. Statistical analysis revealed that sex and age distributions were not significantly different between the two periods (*p* > 0.05). The proportion of patients with odontogenic infections increased significantly from 36.98 (335/906) during the pandemic to 43.93% (311/708) in the post-pandemic period, while the proportion of patients with non-odontogenic infections decreased correspondingly from 63.02 (571/906) to 56.07% (397/708; both *p* < 0.05). The five most common causes of non-odontogenic infections were post-traumatic infections, tonsillitis, sialadenitis, postoperative infections, and iatrogenic infections. However, no significant differences were observed in the distribution of these non-odontogenic etiologies between the two periods (Table 1).

**Table 1:** Baseline characteristics of patients with positive microbial culture [*n* (%)].

Content		Pandemic period (*n* = 906)	Post-pandemic period (*n* = 708)	χ^2^	*P*-value
Sex				0.213	0.644
	Male	567 (62.58)	451 (63.7)		
	Female	339 (37.42)	257 (36.3)		
	Gender ratio (m/f)	1.67	1.75		
Age				0.092	0.762
	0–14 years	63 (6.95)	52 (7.34)		
	>14years	843 (93.05)	656 (92.66)		
	Age (median, IQR)	47 (0.42–89)	49 (0.67–93)		
Etiologic conditions				7.999	0.005^*^
	Odontogenic	335 (36.98)	311 (43.93)		
	Non-odontogenic	571 (63.02)	397 (56.07)		
	Posttraumatic	179 (19.76)	124 (17.51)	1.311	0.252
	Tonsillitis	102 (11.26)	80 (11.30)	0.001	0.979
	Sialadenitis	130 (14.35)	87 (12.29)	1.450	0.229
	Postoperative	97 (10.71)	68 (9.60)	0.526	0.468
	Iatrogenic	50 (5.52)	30 (4.24)	1.385	0.239
	Others	13 (1.42)	8 (1.13)	0.288	0.592

^*^p < 0.05.

### 3.3 Strain composition

In the odontogenic infection group, a total of 1,355 strains were isolated from 646 patients, with an average of 2.10 strains per patient. The microbial profile was predominantly composed of anaerobes, accounting for 65.39% (886/1,355) of isolates, followed by aerobes at 34.32% (465/1,355) and fungi at 0.30% (4/1,355). Throughout the study, the most frequently identified pathogens were *Viridans streptococci* (22.66%, 307/1,355), *P. intermedia* (19.34%, 262/1,355), and *P. acnes* (17.93%, 243/1,355). A comprehensive overview of pathogen identification results is presented in Table 2. Comparative analysis between the two periods revealed that *Propionibacterium* spp. (22.91 vs. 18.40%) and *Actinomyces* spp. (6.20 vs. 2.30%) were more prevalent during the pandemic period, while *Staphylococcus* spp. (1.77 vs. 3.89%) and *Prevotella* spp. (27.22 vs. 33.27%) were less frequently isolated (all *p* < 0.05). Aside from these specific shifts, the pathogen spectrum remained largely consistent between the periods, with the exception of *S. aureus*, which increased significantly from 1.52 to 3.19% (*p* < 0.05).

**Table 2:** Pathogens isolated from patients with odontogenic infections from 2020 to 2024 [*n* (%)].

Microorganisms	Pandemic period			Post-pandemic period		*p*-value
	2020	2021	2022	2023	2024	
Aerobes	88 (32.47)	96 (36.5)	83 (32.42)	95 (33.10)	103 (37.05)	0.634
*Streptococcus* spp.	84 (31.00)	88 (33.46)	62 (24.22)	73 (25.44)	75 (26.98)	0.167
*Viridans streptococcus*	72 (26.57)	60 (22.81)	52 (20.31)	66 (23.00)	57 (20.50)	0.510
*Staphylococcus* spp.	1 (0.37)	2 (0.76)	11 (4.30)	12 (4.18)	10 (3.60)	0.017^*^
*S. aureus*	1 (0.37)	1 (0.38)	10 (3.91)	10 (3.48)	8 (2.88)	0.04^*^
*Klebsiella spp*.	1 (0.37)	4 (1.52)	5 (1.95)	8 (2.79)	7 (2.52)	0.061
Other aerobes bacteria	14 (5.17)	31 (11.79)	16 (6.25)	11 (3.83)	31 (11.15)	-
Anaerobes	182 (67.16)	167 (63.50)	173 (67.58)	189 (65.85)	175 (62.95)	0.529
*Propionibacterium* spp.	66 (24.35)	45 (17.11)	70 (27.34)	44 (15.33)	60 (21.58)	0.045^*^
*P. acnes*	64 (23.62)	43 (16.35)	33 (12.89)	43 (14.98)	60 (21.58)	0.81
*Actinomyces* spp.	20 (7.38)	18 (6.84)	11 (4.30)	2 (0.70)	11 (3.96)	0.001^*^
*A.odontolyticus*	8 (2.95)	11 (4.18)	6 (2.34)	1 (0.35)	10 (3.60)	0.169
*Prevotella* spp.	61 (22.51)	76 (28.90)	78 (30.47)	110 (38.33)	78 (28.06)	0.016^*^
*P.intermedia*	45 (16.61)	34 (12.93)	64 (25)	73 (25.44)	46 (16.55)	0.174
Other anaerobes bacteria	35 (12.92)	28 (10.65)	14 (5.47)	33 (11.50)	26 (9.35)	–
Fungi	1 (0.37)	0 (0.00)	0 (0.00)	3 (1.05)	0 (0.00)	–
*Candida albicans*	1 (0.37)	0 (0.00)	0 (0.00)	3 (1.05)	0 (0.00)	–
*Total*	271 (100.00)	263 (100.00)	256 (100.00)	287 (100.00)	278 (100.00)	–

^*^p < 0.05.

In the non-odontogenic infection group, a total of 2,743 strains were isolated from 968 patients, averaging 2.84 strains per patient. This group was predominated by aerobes, accounting for 94.90% (2,603/2,743) of isolates, while anaerobes and fungi comprised 4.08% (112/2,743) and 1.02% (28/2,743), respectively. The most abundantly isolated pathogens were *K. pneumoniae* (20.56%, 564/2,743), *A. baumannii* (12.18%, 334/2,743), *S. aureus* (11.41%, 313/2,743), and *P. aeruginosa* (10.46%, 287/ 2,743). Detailed identification results for non-odontogenic infections are summarized in Table 3. The isolation rate of *Klebsiella* spp. was significantly higher in the post-pandemic period compared to the pandemic period (27.10 vs. 21.80%), while that of *Pseudomonas* spp. was significantly lower (11.30 vs. 14.40%; both *p* < 0.05). Other major pathogens exhibited no significant changes in distribution, with the exception of *E. coli*, which increased from 1.80 to 3.30% (*p* < 0.05).

**Table 3:** Pathogens isolated from patients with non-odontogenic infections from 2020 to 2024 [*n* (%)].

Microorganisms	Pandemic period			Post-pandemic period		*p*-value
	2020	2021	2022	2023	2024	
Aerobes	269 (90.57)	503 (95.63)	492 (97.04)	666 (94.74)	673 (94.79)	0.744
*S. aureus*	47 (15.82)	57 (10.84)	57 (11.24)	77 (10.95)	75 (10.56)	0.267
*Coagulase-negative staphylococci*	14 (4.72)	25 (4.75)	10 (1.97)	22 (3.13)	28 (3.94)	0.838
other staphylococci	4 (1.35)	3 (0.57)	3 (0.59)	1 (0.14)	8 (1.13)	0.717
*Streptococcus* spp.	10 (3.37)	14 (2.66)	30 (5.92)	24 (3.41)	17 (2.39)	0.097
*Klebsiella* spp.	54 (18.18)	111 (21.10)	125 (24.65)	180 (25.60)	203 (28.59)	0.001^*^
*K. pneumoniae*	50 (16.84)	105 (19.96)	120 (23.67)	152 (21.62)	137 (19.30)	0.885
Other Klebsiella	4 (1.34)	6 (1.14)	5 (0.98)	28 (3.98)	66 (9.29)	0.726
*Escherichia* spp.	35 (11.78)	44 (8.37)	40 (7.89)	79 (11.24)	73 (10.28)	0.112
*E.coli*	6 (2.02)	10 (1.90)	8 (1.58)	22 (3.13)	25 (3.52)	0.012^*^
*Pseudomonas* spp.	45 (15.15)	87 (16.54)	59 (11.64)	78 (11.10)	82 (11.55)	0.017^*^
*Aeruginosa*	41 (13.80)	45 (8.56)	53 (10.45)	74 (10.53)	74 (10.42)	0.984
other Pseudomonas	4 (1.35)	42 (7.98)	6 (1.18)	4 (0.57)	8 (1.13)	–
*Acinetobacter* spp.	32 (10.77)	112 (21.29)	124 (24.46)	144 (20.48)	119 (16.76)	0.308
*A. baumannii*	22 (7.40)	61 (11.60)	64 (12.62)	98 (13.94)	89 (12.54)	0.081
other Acinetobacter	10 (3.37)	51 (9.70)	60 (11.83)	46 (6.54)	30 (4.23)	–
Other Aerobes bacteria	28 (9.43)	50 (9.51)	44 (8.68)	61 (8.68)	68 (9.58)	–
Anaerobes	21 (7.07)	19 (3.61)	15 (2.96)	29 (4.13)	28 (3.94)	0.893
*Prevotella* spp.	8 (2.69)	12 (2.28)	9 (1.78)	18 (2.56)	16 (2.25)	0.693
*Actinomyces* spp.	6 (2.02)	0 (0.00)	3 (0.59)	2 (0.28)	6 (0.85)	0.712
*Propionibacterium* spp.	3 (1.01)	5 (0.95)	3 (0.59)	7 (1.00)	3 (0.42)	0.72
Other anaerobes bacteria	4 (1.35)	2 (0.38)	0 (0.00)	2 (0.28)	3 (0.42)	0.687
Fungi	7 (2.36)	4 (0.76)	0 (0.00)	8 (1.14)	9 (1.27)	0.327
*Candida albicans*	7 (2.36)	4 (0.76)	0 (0.00)	8 (1.14)	9 (1.27)	0.327
Total	297 (100.00)	526 (100.00)	507 (100.00)	703 (100.00)	710 (100.00)	–

^*^p < 0.05.

### 3.4 Detection of multidrug-resistant bacteria

**Figure 2:**
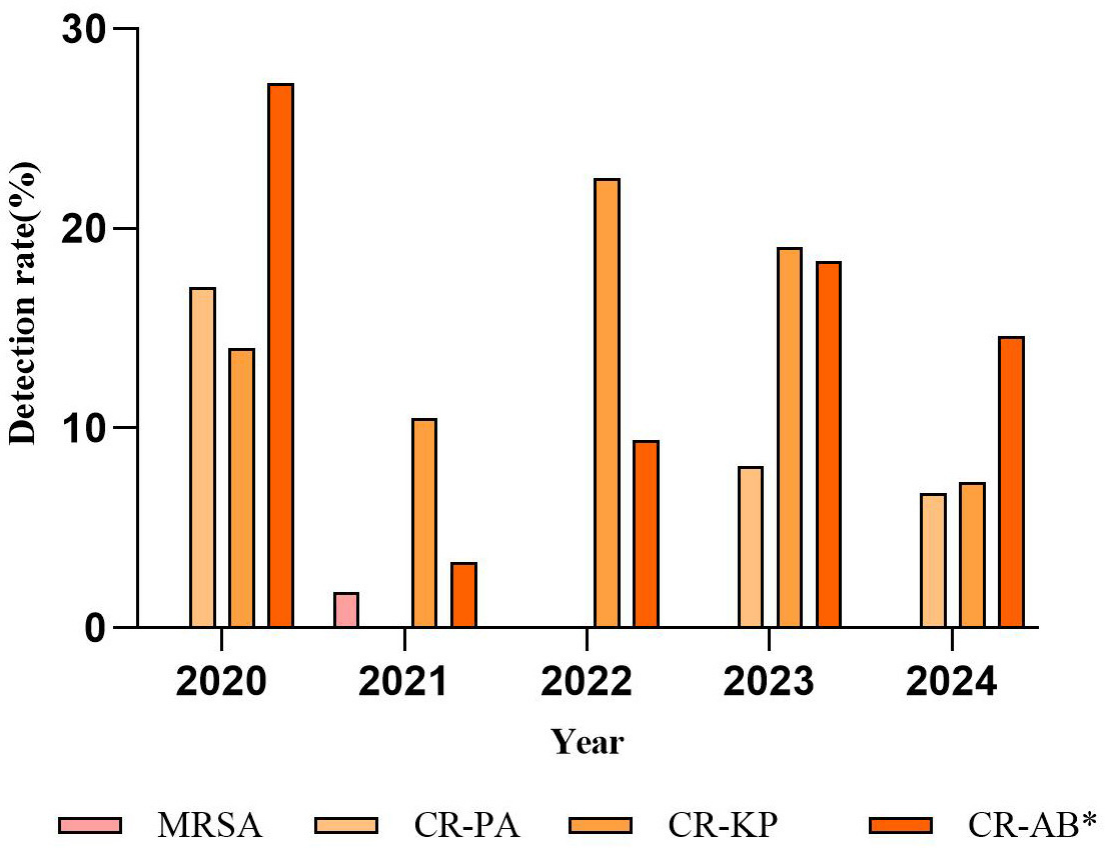
Changes in the detection rate of multidrug-resistant bacteria in non-odontogenic maxillofacial infection, 2020–2024. MRSA, methicillin -resistant *Staphylococcus aureus*; CR-PA, carbapenem-resistant *P. aeruginosa*; CR-KP, carbapenem-resistant *K.pneumoniae*; CR-AB, carbapenem-resistant *A. baumannii*; *p < 0.05.

Throughout the study period, no multidrug-resistant (MDR) bacteria were detected in the odontogenic infection group. In contrast, in the non-odontogenic infection group, the annual numbers of MDR isolates from 2020 to 2024 were 20, 14, 33, 53, and 28, respectively. The MDR strains in this group primarily included CR-AB, carbapenem-resistant *K. pneumoniae* (CR-KP), carbapenem-resistant *P. aeruginosa* (CR-PA), and methicillin-resistant *S. aureus* (MRSA). When comparing the pandemic and post-pandemic periods, the isolation rates of most MDR bacteria remained relatively stable. However, the isolation rate of CR-AB increased significantly from 9.52 (14/147) during the pandemic period to 17.11% (32/187) in the post-pandemic period (*p* < 0.05) (Figure 2).

### 3.5 Resistance of major pathogens in oral and maxillofacial infections to common antimicrobial agents

**Figure 3:**
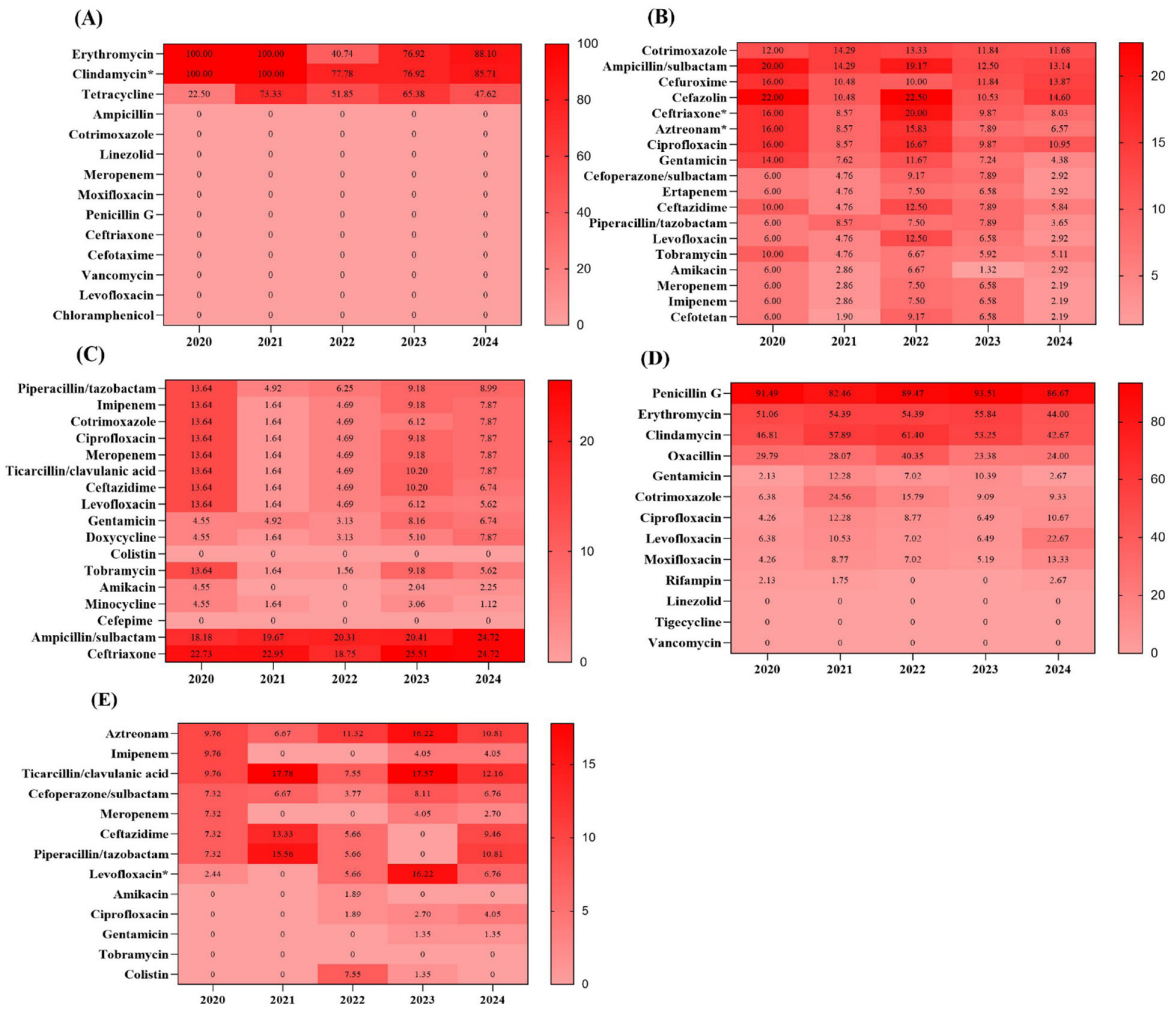
Antimicrobial resistance profiles of *Viridans streptococcus* in odontogenic infection group **(A)**, *K. pneumoniae* **(B)**, *A. baumannii* **(C)**, *S. aureus* **(D)**, and *P.aeruginosa* **(E)** in non-odontogenic infection group isolated from 2020 to 2024. Numbers in the heat maps represented the percentage of antimicrobial resistance. *p < 0.05 when compared to data during and post-pandemic periods.

Over the five-year study period, *Viridans streptococci* isolated from odontogenic infections demonstrated complete susceptibility to several classes of antimicrobial agents, including penicillins, sulfonamides, quinolones, third-generation cephalosporins, and vancomycin. The resistance rate to tetracycline was below 50%, while resistance rates for erythromycin and clindamycin both exceeded 83%. Comparative analysis between the two periods revealed that the resistance rate to clindamycin was significantly higher during the pandemic period (93.81%) than in the post-pandemic period (82.35%; *p* < 0.05). No significant differences were observed for antimicrobial agents other than clindamycin between the two periods (Figure 3A).

For *K. pneumoniae*, the most common pathogen causing non-odontogenic infections, resistance rates varied considerably across different antimicrobial agents in this study. Relatively low resistance rates, ranging from 3 to 9%, were observed for levofloxacin, aminoglycosides, carbapenems, piperacillin/tazobactam, and cefoperazone/sulbactam. In contrast, higher resistance rates exceeding 10% were observed for sulfonamides, ciprofloxacin, aztreonam, and ampicillin/sulbactam. Notably, the resistance rates to ceftriaxone (14.91 vs. 9%) and aztreonam (13.09 vs. 7.27%) were significantly higher during the pandemic period compared to the post-pandemic period (both *p* < 0.05). With the exception of ceftriaxone and aztreonam, resistance rates to other antimicrobial agents remained similar between the two periods (Figure 3B).

*A. baumannii* isolated from non-odontogenic infections showed no resistance to colistin or cefepime throughout the study period. Low resistance rates, both below 2%, were observed for amikacin and minocycline. Moderate resistance levels, ranging from 4 to 9%, were recorded for several classes of antimicrobial agents, including aminoglycosides, quinolones, carbapenems, and piperacillin/tazobactam. In contrast, resistance rates reached higher levels for ampicillin/sulbactam and ceftriaxone, exceeding 20%. Notably, the resistance rates of *A. baumannii* to antimicrobial agents did not exhibit significant variations between the two periods (Figure 3C).

*S. aureus* isolated from non-odontogenic infections exhibited complete susceptibility to linezolid, vancomycin, and tigecycline throughout the study period. Relatively low resistance rates, all below 13%, were observed for gentamicin, rifampin, quinolones, and sulfonamides. In contrast, moderate resistance levels, ranging from 28 to 53%, were recorded for penicillins, clindamycin, and erythromycin. Notably, the resistance rate to penicillin G exceeded 88%. Comparative analysis of resistance rates for all antimicrobial agents revealed no significant differences between the two periods (Figure 3D).

*P. aeruginosa* isolated from non-odontogenic infections showed no resistance to tobramycin throughout the study period. Low resistance rates (<4%) were observed for ciprofloxacin, colistin, aminoglycosides, and carbapenems. In contrast, resistance rates ranging from 6 to 18% were recorded for levofloxacin, ceftazidime, cefoperazone/sulbactam, and piperacillin/tazobactam. Notably, the resistance rate to levofloxacin was significantly lower during the pandemic period than in the post-pandemic period (2.88 vs. 11.49%; *p* < 0.05). With the exception of levofloxacin, the resistance rates of *P.aeruginosa* to antimicrobial agents did not show significant changes between the two periods (Figure 3E).

### 3.6 Correlation between AUD and resistance rate

The annual AUD among patients with oral and maxillofacial infections ranged from 28.38 to 34.85 DDDs per 100 patient-days from 2020 to 2024. No statistically significant difference in AUD was observed between the two periods (*p* > 0.05). Similarly, the AUD for meropenem showed an upward but non-significant trend (*p* > 0.05) (Table 4). Furthermore, no significant correlation was found between meropenem use density and the meropenem resistance rates of *A. baumannii, K. pneumoniae*, or *P. aeruginosa* in the non-odontogenic infection group (*p* > 0.05) (Table 5).

**Table 4:** Comparison of AUD from 2020 to 2024 (DDDs/100 patient-days).

Antibiotic agents	Pandemic period			Post-pandemic period		*p*-value
	2020	2021	2022	2023	2024	
All antimicrobial	34.11	31.27	28.38	31.87	34.85	0.248
Meropenem	0.32	0.25	0.28	0.49	0.48	0.083

**Table 5:** Correlation between meropenem use density and resistance rates to meropenem in *A.baumannii, K. pneumoniae*, and *P.aeruginosa*.

Antimicrobial resistance rate	R	*p*-value
*A.baumannii*-meropenem	0.7	0.188
*K. pneumoniae*-meropenem	0	1
*P.aeruginosa*-meropenem	0.667	0.219

## 4 Discussion

The microbial culture submission rate remained stable between the two periods in this study, indicating that clinical criteria for initiating microbial investigations were largely consistent. However, the lack of national surveillance data on oral and maxillofacial infections in China prevented a direct comparison of our positivity rates with national benchmarks. Notably, the culture positivity rate in this study was lower than the historical benchmark of 74.5% previously reported by our hospital, a discrepancy that underscores the influence of varying case severity on culture positivity rates (18). Previous studies predominantly focused on patients with severe OMSI, in whom microbial load in deep pus specimens was typically high. In contrast, the present study encompassed a broader clinical spectrum, including mild and moderate cases, which may explain the relatively lower positivity rate. Furthermore, the positivity rate decreased from 20.19 during the pandemic to 18.71% in the post-pandemic period. This decline is likely influenced by multiple confounding factors, such as prior antibiotic use, previous maxillofacial treatment, and the duration of infection. Previous studies from our institution have reported that from 2003 to 2018, 22.3%−48.1% of patients had used antibiotics or received maxillofacial treatment before admission, and 57% of patients had a shorter disease duration (18, 19). During the pandemic, reduced access to antibiotics and delayed medical intervention may have increased the risk of infection progression; in contrast, the restoration of medical services in the post-pandemic period likely led to a higher proportion of patients presenting with a history of self-medication with antibiotics or prior outpatient treatments. These factors may have inhibited bacterial growth in clinical specimens, contributing to the observed decline in the microbial culture positivity rate.

Among patients with positive microbial cultures, a predominance of males, adults, and non-odontogenic infections was observed, with post-traumatic infection being the most common etiology. This finding contrasts with international studies that often report tonsillitis or lymphadenitis as leading causes (2, 5). The discrepancy may be attributed to our hospital's role as the only stomatology hospital in Southwest China with a dedicated maxillofacial trauma ward, which likely skews the case composition toward trauma-related etiologies. Although the distribution of non-odontogenic etiologies remained stable between the two periods, a significant shift was observed in the overall infection landscape: the relative proportion of odontogenic infections was notably lower during the pandemic period than in the post-pandemic period. This trend diverges from existing reports that have documented an increase in admissions for odontogenic infections during the pandemic period (20–23). This disparity likely arises from the pandemic-induced restriction of routine dental services, which hindered timely professional intervention for odontogenic infections; conversely, emergency admission pathways for trauma-related infections were prioritized and remained operational (24–28). Notably, previous research has consistently demonstrated that both odontogenic and non-odontogenic infections can lead to severe complications (6, 29). Consequently, prompt initiation of targeted antimicrobial therapy remains essential for preventing adverse outcomes.

Odontogenic infections are polymicrobial, involving various aerobic bacteria, anaerobic bacteria, and fungi, most of which are part of the normal oral flora of the host (30–33). While early stages of infection are often dominated by *Streptococcus* spp., chronic progression shifts the microbial profile toward anaerobe predominance (34). Consistent with global retrospective analyses (35), our study found that anaerobic isolation rates exceeded those of aerobes (36). Specifically, *Streptococcus* spp. were the most frequently isolated organisms, followed by *Prevotella* spp. and *Propionibacterium* spp., mirroring patterns reported by the German Antimicrobial Resistance Surveillance system (17, 37–39). In contrast to some international reports, *Staphylococcus* spp. were not among the primary pathogens in this cohort, a finding consistent with previous data from our institution (18). These variations likely stem from differences in geographic, economic, healthcare infrastructure, and prescribing practice factors (35). Furthermore, we observed significant changes in the isolation rates of *Staphylococcus* spp., *Propionibacterium* spp., *Actinomyces* spp., and *Prevotella* spp. between the two periods, likely influenced by pandemic-related healthcare strategies and altered antibiotic availability (38). From a clinical perspective, an increased prevalence of *Staphylococcus* spp. could signal more virulent infections and pose greater management challenges (40), although their precise role, whether transient commensal or definitive pathogens, remains unclear (35, 41).

Although the isolation rate of *Viridans streptococci* at our hospital remained stable between the two periods, the observed AMR patterns necessitate rigorous evaluation to optimize empirical treatment and improve clinical outcomes. While resistance to clindamycin showed a slight decline from 93.81 to 82.35%, it remained persistently elevated, consistently exceeding 80%. This finding contrasts sharply with previous reports, in which clindamycin resistance typically remained below 20% (17, 42). This discrepancy is directly linked to the historical over-reliance on clindamycin in domestic dental practice, which often deviates from Chinese clinical guidelines that restrict the drug's use to patients with confirmed allergies to penicillins or cephalosporins (43). Historically, the misinterpretation of false-positive results during β-lactam skin testing may have precipitated the inappropriate overuse of clindamycin as a second-line alternative. However, following the National Health Commission's 2021 implementation of the “Guidelines for Skin Tests of β-Lactam Antimicrobial Drugs,” the standardization of these diagnostic procedures is expected to improve antimicrobial stewardship. In contrast, the isolates in this study remained fully sensitive to recommended first-line antibiotics, such as penicillin and aminopenicillins. This sustained susceptibility is likely attributable to the absence of β-lactamase production and the lack of significant penicillin-binding protein mutations in the local strains. Collectively, these findings indicate that clindamycin should be strictly regulated in the management of odontogenic infections, while penicillin-based antibiotics should remain the primary therapeutic choice.

An imbalance of oral microbial ecology is a key factor driving the proliferation of opportunistic pathogens and the subsequent development of non-odontogenic infections. Common opportunistic pathogens in these cases include aerobic Gram-positive cocci (*S. aureus* and β*-hemolytic Streptococcus*) and aerobic Gram-negative bacilli (*E. coli, K. pneumoniae, A. baumannii*, and *P. aeruginosa*). Compared with odontogenic infections, the pathogen spectrum of non-odontogenic infections exhibits greater specificity, as the microbial composition may be influenced by the primary etiology and the local microenvironment (44). While previous studies have predominantly identified aerobic Gram-positive cocci as the primary causative agents, the present study found aerobic Gram-negative bacilli to be dominant. This discrepancy may be attributed to the predominance of trauma-related infections in this study (2, 5). Generally, non-odontogenic infections stemming from tonsillitis or lymphadenitis are frequently associated with *S. aureus* and β*-hemolytic Streptococcus*, whereas trauma-related infections are more susceptible to trauma environmental microorganisms, with common pathogens including *K. pneumoniae, A. baumannii*, and *P. aeruginosa* (5, 45).

In this study, aerobic Gram-negative bacilli accounted for approximately 66% of isolates from non-odontogenic infections, a finding consistent with the CHINET national wound pus pathogen monitoring report from 2015 to 2021 (46). The most common pathogens identified were *K. pneumoniae*, followed by *A. baumannii, S. aureus*, and *P. aeruginosa*. This species composition aligns closely with the aforementioned national monitoring report, with only minor variations in rank order. Notably, the isolation rates of these major pathogens exhibited no significant fluctuations during the study period. This stability likely reflects the relatively consistent distribution of primary etiologies associated with non-odontogenic infections over time. Furthermore, although an upward trend was observed in the isolation rate of *E. coli*, the limited sample size suggests that this finding requires validation through prospective, multi-center studies with larger cohorts.

AMR among these opportunistic pathogens is influenced by multiple factors, including institutional antibiotic prescribing practices and regional resistance patterns. The development of resistance is further exacerbated by both the long-term, non-standard use of antibiotics within hospital settings and the widespread deployment of broad-spectrum antimicrobial agents at the national level, particularly during the pandemic period. These factors collectively contribute to elevated resistance levels in pathogens and consequently increase the risk of treatment failure.

*K. pneumoniae* is an opportunistic pathogen that frequently colonizes the oropharyngeal mucosa. Upon breaching the mucosal barrier, it can lead to severe and potentially life-threatening infections, which are often characterized by high virulence and extensive drug resistance (34). In this study, resistance rates to ceftriaxone remained relatively high (>10%) during the pandemic period, while lower resistance levels were observed for cefoperazone/sulbactam and piperacillin /tazobactam. Although these resistance levels were below national averages (>30%), our institution had implemented stringent antimicrobial stewardship measures (47, 48). Consequently, a significant reduction in ceftriaxone resistance rates was observed in the post-pandemic period, a trend likely attributable to these strategic interventions.

Infections caused by *A. baumannii* are associated with a mortality rate ranging from 2 to 10% (49). Traditionally considered a nosocomial pathogen, *A. baumannii* is increasingly recognized for its significance in oral settings, with recent evidence indicating its ability to transition into an oral colonizer (50). Although its role as an oral pathogen remains to be fully elucidated, its capacity for biofilm formation and extensive AMR poses significant therapeutic challenges. These factors contribute to its persistence, as evidenced in this study by persistently high resistance rates (>20%) to ceftriaxone and ampicillin/sulbactam despite the implementation of infection control measures. This finding underscores the need for enhanced antimicrobial stewardship, as biofilm-mediated survival and resistance patterns complicate treatment outcomes.

Our data revealed that the resistance rates of *P. aeruginosa* were markedly lower than national averages (47, 48). Notably, the resistance rate to levofloxacin increased significantly to 11.49% in the post-pandemic period. As the only available quinolone agent in our hospital, levofloxacin is frequently used as an alternative for patients with β-lactam allergies, and this widespread use may have contributed to the observed increase in resistance. To preserve the clinical efficacy of such agents, strategies are required to strengthen the clinical management of antibacterial drugs.

As expected, *S. aureus* resistance rates to penicillin and clindamycin remained high (>42%), and an upward trend was also observed for levofloxacin resistance. Given its prevalence as a key pathogen in non-odontogenic infections, such high levels of resistance to these common antimicrobial agents pose significant challenges to effective clinical treatment. Notably, only one MRSA strain was identified during the study period, and all *S. aureus* isolates remained fully susceptible to linezolid and vancomycin, which continue to be critical alternative options for clinical therapy.

Currently, resistance of Gram-negative bacilli to carbapenems has become a serious global public health challenge (51). This study demonstrated that while the overall carbapenem resistance rates of *K. pneumoniae, A. baumannii*, and *P. aeruginosa* remained at relatively low levels (2%−7%), they exhibited distinct dynamic fluctuations. The AUD of meropenem, the only available carbapenem agent utilized in our hospital, exhibited a marginal increase, though this had a limited impact on the corresponding resistance rates. Such fluctuations may be influenced by multiple factors, including the specific AUD of carbapenems, the selection of particular carbapenem agents, and the effectiveness of hospital infection control measures over time (52, 53). Furthermore, the detection rate of MDR bacteria in this study was relatively stable. However, consistent with national trends, the detection rate of CR-KP remained at a high level (>13%), while the detection rate of CR-AB significantly increased to 17.11% (54). Consequently, in response to these concerning trends, it is imperative to implement targeted and continuous hospital infection control measures to mitigate the transmission of resistant strains and reduce the risk of associated infections.

This retrospective, single-center study has several limitations that should be considered when interpreting the findings. First, the single-center design restricts the generalizability of the results. Although we adjusted for age, sex, and primary etiology, other confounding factors may persist, such as differences in antimicrobial stewardship policies, alterations in prescribing practices, and changes in patient healthcare-seeking behavior. As these unmeasured variables may independently influence microbiological patterns, a direct causal relationship between COVID-19 infection and the observed changes could not be definitively established. Furthermore, the relatively small number of isolates available for analysis in certain subgroups limits the statistical significance of the shifts in antimicrobial resistance rates. Therefore, future research should adopt multi-center prospective designs with larger sample sizes, longer follow-up periods, and rigorous adjustment for potential confounders to enhance the robustness and reliability of the findings.

## Data Availability

The original contributions presented in the study are included in the article/supplementary material, further inquiries can be directed to the corresponding authors.
